# Supplementary material for: Tracing PFAS Transfer from Mother to the Fetoplacental Unit: Insights from Trimester-Specific Maternal Serum Profiles
Source: bioRxiv. 2026 Jun 24:2026.02.02.703409. Preprint. [Version 2] doi: 10.64898/2026.02.02.703409 (PMC13320966; doi:10.64898/2026.02.02.703409)
Supplement: Supplement 8 [file NIHPP2026.02.02.703409v2-supplement-8.pdf]

bioRxiv preprint doi: https://doi.org/10.64898/2026.02.02.703409; this version posted June 24, 2026. The copyright holder for this preprint (which was not certified by peer review) is the author/funder, who has granted bioRxiv a license to display the preprint in perpetuity. It is made available under a CC-BY 4.0 International license.

SUPPLEMENTARY TABLES

Supplementary Table 1 Above limit-of-detection rates for the PFAS studied.

Supplementary Table 2 Case complete placental included vs. excluded comparison.

Supplementary Table 3 Case complete cord blood included vs. excluded comparison.

Supplementary Table 4 Model effect estimates for placental PFAS (ng/g). Effect estimates correspond to a doubling in maternal timepoint PFAS concentration (μg/L).

Supplementary Table 5 Stochastic intervention g-formula model effect estimates for placental PFAS (ng/g). Effect estimates correspond to an additive shift of 0.25 standard deviations on the original scale (μg/L) pooled across maternal serum measures.

Supplementary Table 6 Model effect estimates for cord plasma PFAS (μg/L). Effect estimates correspond to a doubling in maternal timepoint PFAS concentration (μg/L) or placental PFAS concentration (ng/g).

Supplementary Table 7 Stochastic intervention g-formula model effect estimates for cord blood serum PFAS (μg/L). Effect estimates correspond to an additive shift of 0.25 standard deviations on the original scale (μg/L or ng/g) pooled across maternal serum measures.

bioRxiv preprint doi: https://doi.org/10.64898/2026.02.02.703409; this version posted June 24, 2026. The copyright holder for this preprint (which was not certified by peer review) is the author/funder, who has granted bioRxiv a license to display the preprint in perpetuity. It is made available under a CC-BY 4.0 International license.

SUPPLEMENTARY FIGURES

Supplementary Figure 1 Directed acyclic graph describing the longitudinal parametric g-formula for placental PFAS as the outcome. L0 describes the vector of fixed baseline covariates: maternal body mass index, maternal age, maternal education and fetal sex. A describes maternal serum PFAS level at each timepoint (indicated with _1, _2, _3). Y describes placental PFAS level. L1 represents time-varying confounders measured at the same timepoints as maternal exposure. We tested three sensitivity models that considered L1 albumin serum level, estimated glomerular filtration rate, or maternal weight.

Supplementary Figure 2 Directed acyclic graph describing the longitudinal parametric g-formula for cord blood serum as the outcome. L0 describes the vector of fixed baseline covariates: maternal body mass index, maternal age, maternal education and fetal sex. A describes maternal serum PFAS level at each timepoint (indicated with _1, _2, _3). M describes placental PFAS level. Y describes cord blood serum PFAS level. L1 represents time-varying confounders measured at the same timepoints as maternal exposure. We tested three sensitivity models that considered L1 albumin serum level, estimated glomerular filtration rate, or maternal weight.

Supplementary Figure 3 Spearman correlation matrix comparing PFAS measures across biomatrices and timepoints. An X indicates no statistically significant bivariate correlation ($p<0.05$).

Supplementary Figure 4 Stochastic interventions estimating the mean difference in placental concentration corresponding to a 25% standard deviation increase (shift) at one timepoint during pregnancy via the parametric g-formula with time-varying confounders albumin, estimated glomerular filtration rate (eGFR), maternal weight, or none (Basic).

Supplementary Figure 5 Stochastic interventions estimating the mean difference in cord blood serum concentration corresponding to a 25% standard deviation increase (shift) at one timepoint during pregnancy via the parametric g-formula with time-varying confounders albumin, estimated glomerular filtration rate (eGFR), maternal weight, or none (Basic).

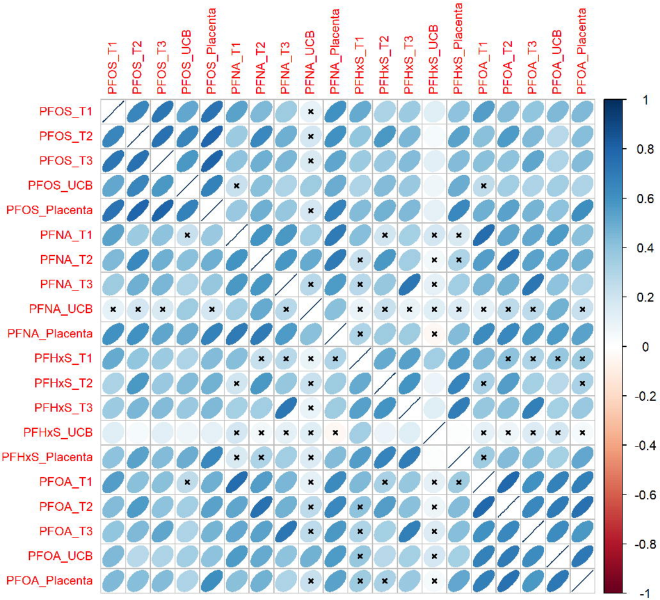
PFOS_T1
PFOS_T2
PFOS_T3
PFOS_UCB
PFOS_Placenta
PFNA_T1
PFNA_T2
PFNA_T3
PFNA_UCB
PFNA_Placenta
PFHxS_T1
PFHxS_T2
PFHxS_T3
PFHxS_UCB
PFHxS_Placenta
PFOA_T1
PFOA_T2
PFOA_T3
PFOA_UCB
PFOA_Placenta
1
0.8
0.6
0.4
0.2
0
-0.2
-0.4
-0.6
-0.8
-1

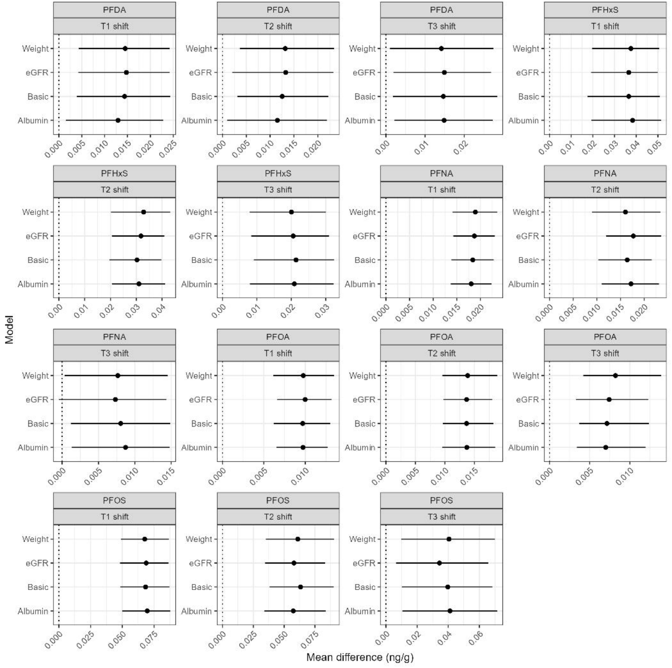
PFDA
PFHxS
PFNA
PFOA
PFOS
T1 shift
T2 shift
T3 shift
Weight
eGFR
Basic
Albumin
Model
Mean difference (ng/g)

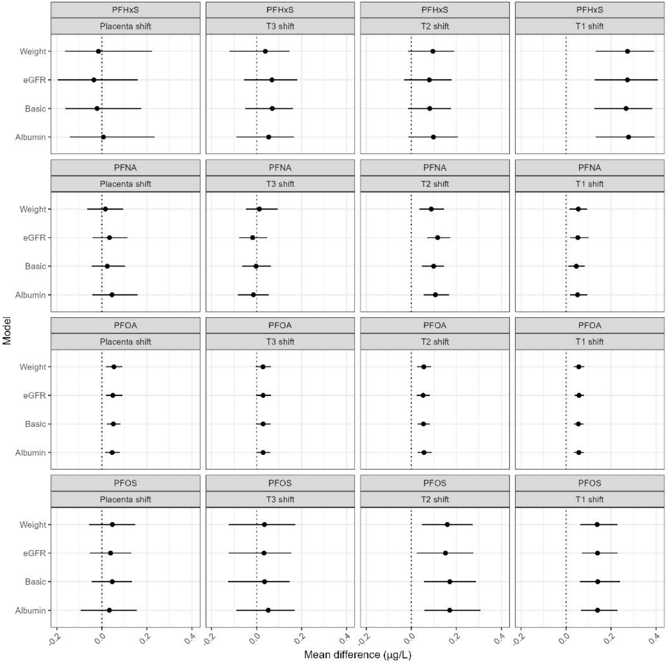

PFHxS
PFNA
PFOA
PFOS
Placenta shift
T3 shift
T2 shift
T1 shift
Weight
eGFR
Basic
Albumin
Model
-0.2
0.0
0.2
0.4
Mean difference (µg/L)

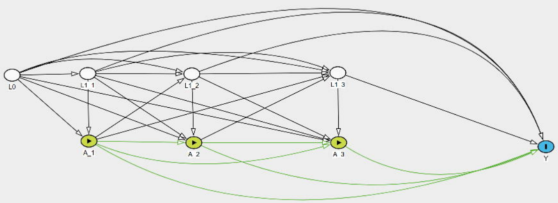

L0
L1_1
L1_2
L1_3
A_1
A_2
A_3
Y

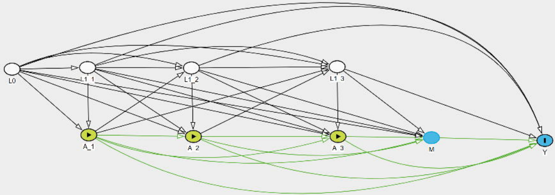
L0
L1_1
L1_2
L1_3
A_1
A_2
A_3
M
Y
